# A new model for efficient, need‐driven progress in generating primary biodiversity information resources

**DOI:** 10.1002/aps3.11318

**Published:** 2020-01-19

**Authors:** Alex Asase, Moses N. Sainge, Raoufou A. Radji, Omokafe A. Ugbogu, A. Townsend Peterson

**Affiliations:** ^1^ Department of Plant and Environmental Biology University of Ghana P.O. Box LG 55 Legon Ghana; ^2^ Tropical Plant Exploration Group (TroPEG) P.O. Box 18 Mundemba Cameroon; ^3^ Department of Botany University of Lome 06 BP 6135 Lome Togo; ^4^ Forestry Research Institute of Nigeria PMB 5054, Jericho Hills Ibadan Nigeria; ^5^ Biodiversity Institute, and Department of Ecology and Evolutionary Biology University of Kansas Lawrence Kansas USA

**Keywords:** biodiversity informatics, digitization, plants, primary biodiversity data, West Africa

## Abstract

**Premise:**

The field of biodiversity informatics has developed rapidly in recent years, with broad availability of large‐scale information resources. However, online biodiversity information is biased spatially as a result of slow and uneven capture and digitization of existing data resources. The West African Plants Initiative approach to data capture is a prototype of a novel solution to the problems of the traditional model, in which the institutional “owner” of the specimens is responsible for digital capture of associated data.

**Methods:**

We developed customized workflows for data capture in formats directly and permanently useful to the “owner” herbarium, and digitized significant numbers of new biodiversity records, adding to the information available for the plants of the region.

**Results:**

In all, 190,953 records of species in 1965 genera and 331 families were captured by mid‐2018. These data records covered 16 West African countries, with most of the records (10,000–99,999) from Côte d'Ivoire, Ghana, Togo, Nigeria, and Cameroon, and the fewest data records from Mauritania (<100 records). The West African Plants Initiative has increased available digital accessible knowledge records for West African plants by about 54%. Several of the project institutions have put initial project data online as part of their Global Biodiversity Information Facility data contributions. The average cost of data capture ranged from US$0.50−1.00 per herbarium sheet.

**Discussion:**

Data capture has been cost‐effective because it is much less expensive than de novo field collections, allows for development of information resources even for regions in which political situations make contemporary field sampling impossible, and provides a historical baseline against which to compare newer data as they become available. This new paradigm in specimen digitization has considerable promise to accelerate and improve the process of generating high‐quality biodiversity information, and can be replicated and applied in many biodiversity‐rich, information‐poor regions to remedy the present massive gaps in information availability.

Biological diversity is distributed unevenly worldwide, with well‐known biogeographic patterns such as the latitudinal diversity gradient (Mannion et al., 2014), in which the tropics hold many more species than higher‐latitude regions. Other examples of such uneven distribution of biological diversity include the existence of centers of endemism and so‐called “hotspots” of biodiversity (Myers et al., 2000). These imbalances are, for whatever reasons, often correlated (if not causally) with differences in wealth, in which the wealthy, so‐called Global North is relatively biodiversity‐poor, whereas the so‐called Global South is often biodiversity‐rich and economically poor (Balmford et al., 2005; Rands et al., 2010). A third dimension of this equation centers on the geographic distribution of biodiversity data worldwide. As with global wealth, this commodity tends to be concentrated in the Global North and to be relatively sparse and rare in tropical countries. The reasons behind this unequal distribution of information have to do with histories of occupation and colonization (Peterson et al., 2016a), as well as with histories of science and its development largely in the Global North, at least until relatively recently (Collen et al., 2008).

The field of biodiversity informatics has developed rapidly in recent years, with the appearance of important and exciting new tools for analyzing and interpreting biodiversity data. These tools, when linked properly to information availability, have in turn yielded exciting advances in understanding key aspects and dimensions of biodiversity: its distribution in space and time and with respect to environments, and optimal strategies for its conservation (Peterson et al., 2010). Another area of massive recent advance in biodiversity informatics has been in the broad availability of large‐scale information resources such as the Global Biodiversity Information Facility (GBIF; https://www.gbif.org), VertNet (http://vertnet.org), speciesLink (http://splink.cria.org.br), REMIB (http://remib.org), iDigBio (http://www.idigbio.org), and others.

However, online biodiversity information is notoriously and dramatically biased spatially, as has been demonstrated in numerous recent studies (Yesson et al., 2007; Boakes et al., 2010; Stropp et al., 2016; Sosef et al., 2017), which aligns with the information inequities enumerated above. Underdevelopment of biodiversity information resources emerges as a result of slow capture and digitization of existing data resources on the part of the individuals and institutions holding those data and/or specimens; this is a common problem, as the systematics enterprise has become budget stressed and resource starved (Bini et al., 2006). Unfortunately, these processes are long term in nature, and there is no evidence that they are in the process of being resolved.

This paper offers a first view of a new model designed specifically to remedy these imbalances. We noted striking contrasts between the enthusiasm for an entry into the world of biodiversity informatics among scientists from across West Africa, and the information resources that were so painfully slow to appear to support and nourish that enthusiasm. Rather than languish or work with too‐small data resources while awaiting digitization efforts on the part of the institutions that hold the key specimens, we designed an alternative approach, building on previous explorations by Mexican institutions (Soberón and Llorente‐Bousquets, 1993; Sarukhán et al., 2014). Specifically, the model explored herein is one in which scientists in the region can take charge of their own information destinies, and initiate and implement steps to create new information resources.

## THE WEST AFRICAN PLANTS INITIATIVE

The West African Plants (WAP) Initiative is a consortium of West African researchers in botany; its goal is to digitize and mobilize available, high‐quality, primary biodiversity occurrence data resources for West African plant diversity. The project covers a region extending from Senegal and Gambia east to Cameroon and north to Mali, and aims to create a rich data resource that can be made openly available and brought into currency for scientific research, policy decisions, and ultimately for sustainable development. A further goal of the initiative is to develop relevant human resources and skills regarding the plants of West Africa. The consortium includes partners from four West African institutions (University of Ghana, University of Lomé [Togo], Forestry Research Institute of Nigeria, and Tropical Plant Exploration Group [Cameroon]), in coordination with six institutions across Europe and North America (Naturalis Biodiversity Center [The Netherlands]; Royal Botanic Gardens, Kew and World Museum [United Kingdom]; Muséum National d'Histoire Naturelle [France]; and the Missouri Botanical Garden and New York Botanical Garden [United States]). All 10 institutions participating in the project have significant West African botanical holdings in the form of herbarium sheets in numbers ranging from 10,000 to 160,000 or more specimens.

Within both West Africa and Africa more generally, major efforts to digitize and mobilize available primary biodiversity information resources have taken place. For example, the Southern African Botanical Diversity Network (SABONET) project involved computerization of data on labels of herbarium specimens of major herbaria in southern Africa (Siebert and Smith, 2004). The African Plants Initiave (API) project also digitized images of type specimens and associated information on African flora in herbaria in both the Northern and Southern Hemispheres, and made these accessible via electronic and other means for scholarly use (Smith, 2004). These previous efforts have been primarily taxonomic initiatives; WAP, on the other hand, is a new initiative with a different motivation and approach that is primarily focused on biodiversity informatics.

## METHODS

The project linked caretakers of specimens at institutions on three continents with scientists from across West Africa who had an intense interest in accessing data on plants of their region. The West African proponents have the manpower and motivation, whereas European and North American participants increasingly share the desire for data associated with their collections to be digitized and shared globally. In the first phases of this project, we adopted previous workflows for digitization of biological collections (e.g., Nelson et al., 2012; Thiers et al., 2016) with specific modifications to capture data in formats that would be directly and permanently useful to the “owner” herbaria. Data capture started in 2015 and took place only at West African institutions. All data capture was strictly in accordance with DarwinCore standards (Wieczorek et al., 2012), achieved either by (a) capturing data records from existing images (e.g., images supplied by the Naturalis Biodiversity Center at this pilot phase) or (b) capturing data from images taken quickly and efficiently by project interns in West African herbaria. These images were captured using a Bencher BECM2PF Copymate II Fluorescent Tabletop Producer (110‐240V; Bencher Inc., Antioch, Illinois, USA) via a system that controlled for image quality and reduced time spent in post‐imaging batch processing and light adjustment. Data capture was achieved using BRAHMS database software (http://herbaria.plants.ox.ac.uk/bol/), customized for this project. Data capture was carried out by graduate students and technicians trained by this project who were involved at various stages in the project as interns. Selection of plant groups for digitization was guided by the specific interests of the West African partners.

This general model—that data be captured, improved, and mobilized by those who wish to have access to the data, rather than by the institutions holding the collections—is probably particularly needed in some regions of the world. As an initial step toward identifying such regions, we explored the 57,586,379 records of the kingdom Plantae that are based on preserved specimens (i.e., herbarium sheets) and that are served via the GBIF data portal (Global Biodiversity Information Facility, 2017a); this work is preliminary and is part of a broader exploration of the networks of providers and users that characterize the biodiversity informatics world at present (Peterson et al., in prep.). We processed these data from raw numbers in each country to densities of records per km^2^ and then calculated the proportion of the total number of records in each country that are provided by institutions in other countries. This metric can be taken as a measure of dependency of a country on external data sources (percent external) and a measure of overall need for data (record density). We calculated the ratio of the two as a measure of how much particular countries may profitably look to data held in other countries as sources of rich biodiversity data resources. We do not propose this index as a prioritization or explicit ranking, but rather an exploration for consideration and discussion. Indeed, it is rather similar to the “Biodiversity Informatics Potential Index” (Ariño et al., 2011), which was based on (a) the biological richness or ecological diversity of a country, (b) the capacity of the country to generate biodiversity data records, and (c) the availability of technical infrastructure in the country for managing and publishing such records. Rather than classifying countries as having low or high bioinformatics potential, we instead look to guide countries as to which paths to explore in developing their own biodiversity information resources efficiently.

## RESULTS

**Figure 1:**
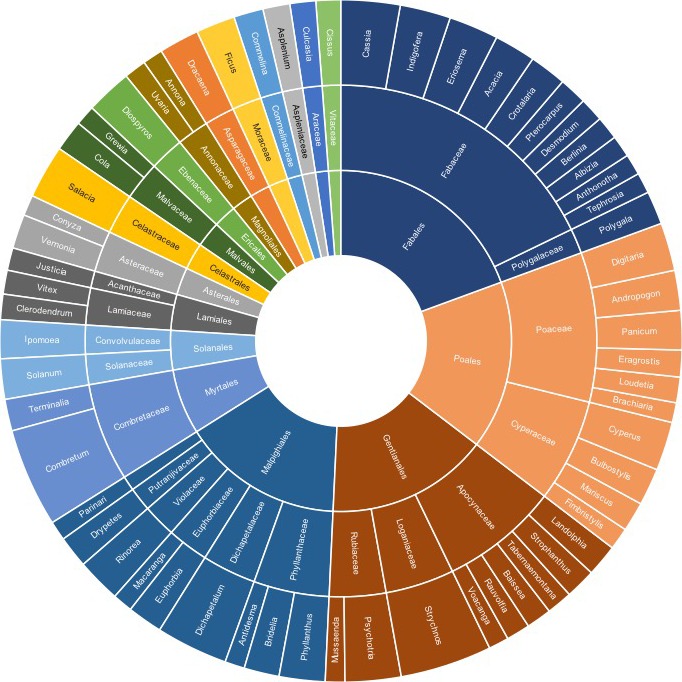
Summary of hierarchical taxonomic representation (order, family, genus) of plant genera among data generated as part of the West African Plants Initiative. Note that we have included in this summary only those genera with ≥500 records. Taxonomic authority is from IRMNG (Rees et al., 2017).

**Figure 2:**
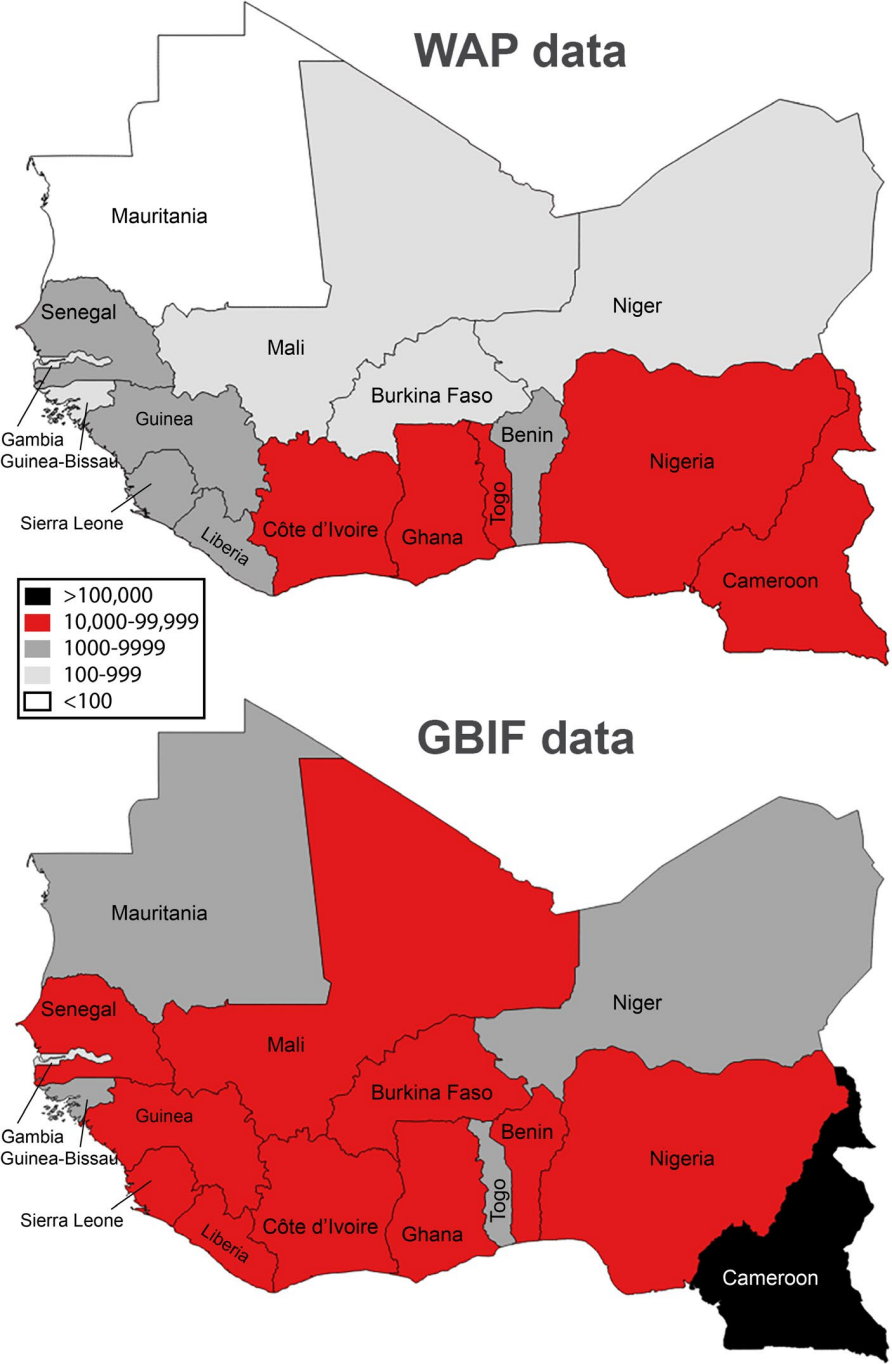
Summary of numbers of plant primary biodiversity data records captured as part of the West African Plants (WAP) Initiative, as compared to data otherwise available already via the Global Biodiversity Information Facility (GBIF).

**Figure 3:**
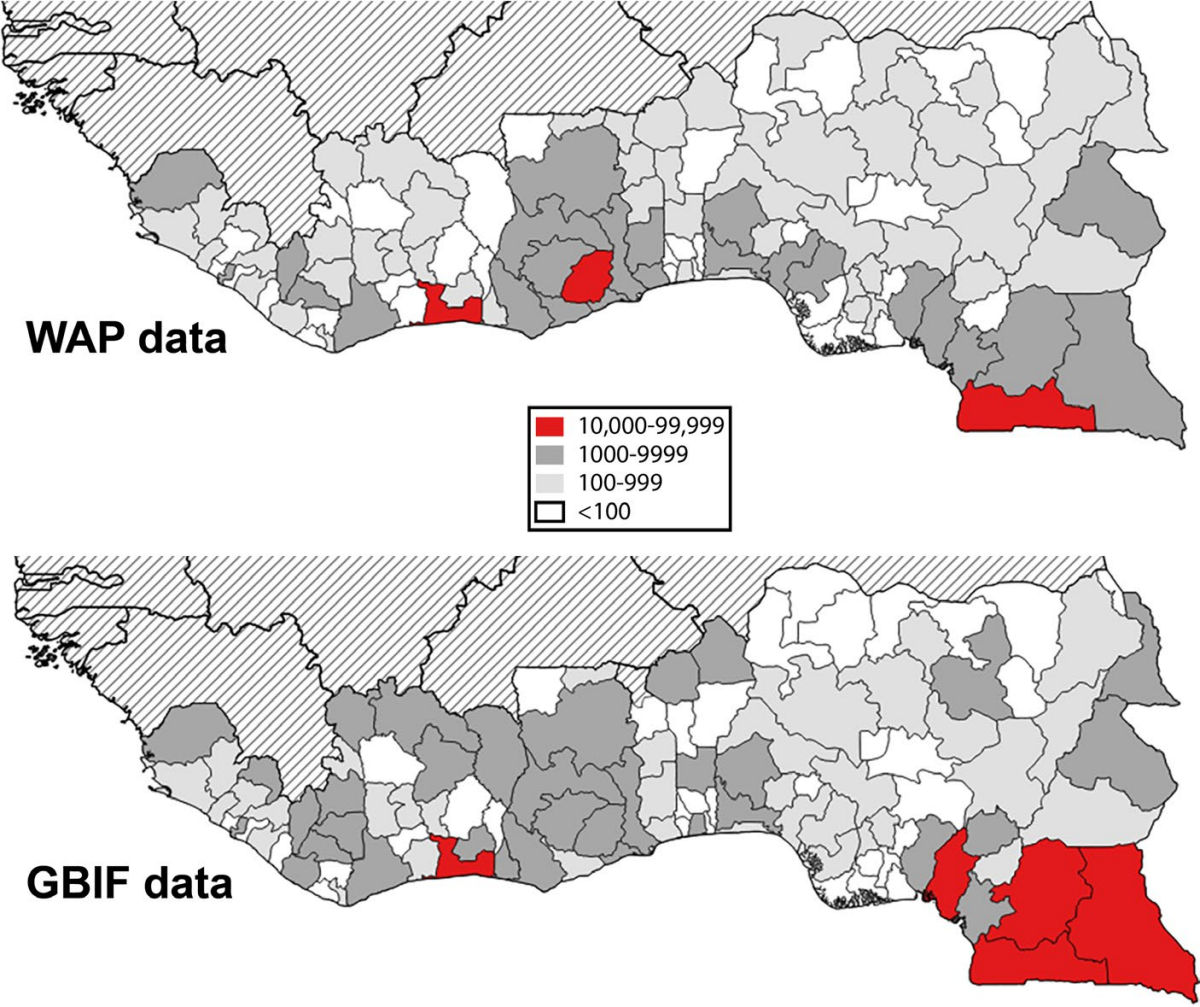
Distribution of primary biodiversity data records generated by the West African Plants (WAP) Initiative, as compared to data otherwise available already via the Global Biodiversity Information Facility (GBIF), at the level of states and provinces across eight West African countries. Surrounding countries all had state‐level record richness <100, and so are not included in this summary.

**Figure 4:**
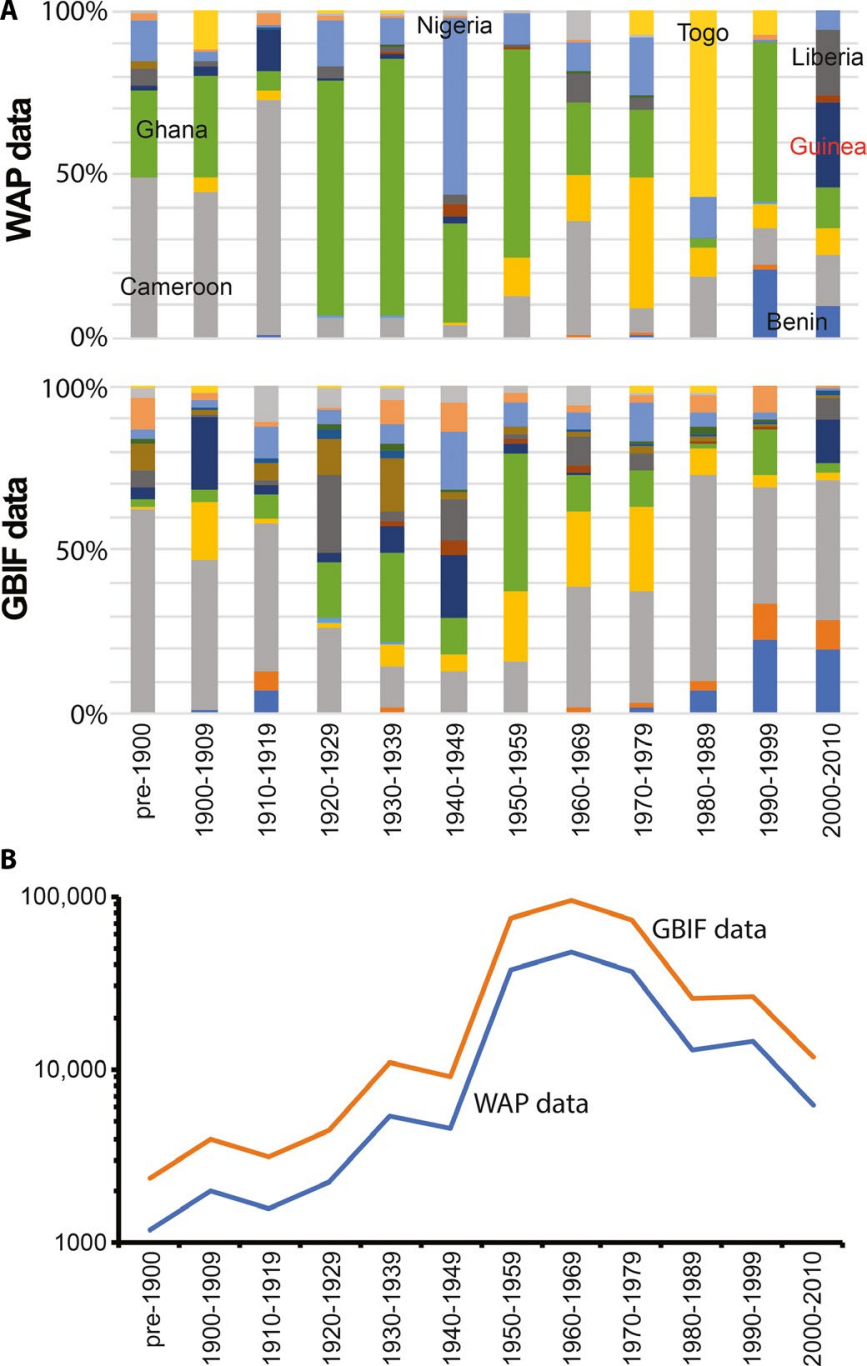
Temporal trends in West African Plants (WAP) Initiative data and Global Biodiversity Information Facility (GBIF) data. (A) Proportional representation of eight West African countries over the period 1900–2009. Country labels indicate which color pertains to which country. (B) Number of records through time, showing year‐to‐year variation. Note that the number of records is shown on a log_10_ scale.

In all, 190,953 records of species in 1965 genera and 331 families were captured by mid‐2018. Hierarchical taxonomic representation (i.e., order, family, genus) of the most common plant genera (i.e., with ≥500 records) among data records generated is summarized in Fig. 1. These primary biodiversity data records covered 16 West African countries, with most of the records (10,000–99,999) from Côte d'Ivoire, Ghana, Togo, Nigeria, and Cameroon, and the fewest data records from Mauritania (<100 records) (Fig. 2). These data records can be tied to subnational regions like states, departments, and provinces, reflecting broad geographic coverage by WAP data resources, but again with gaps and foci that limit the utility of the data (Fig. 3). The temporal distribution of the data records showed the earliest specimens in the first years of the 19th century and a peak in collections activity in the latter half of the 20th century (Fig. 4). Different countries saw increased or reduced collections activity over the centuries, probably as a result of a combination of political situations and collections priorities and interests (Fig. 4).

**Figure 5:**
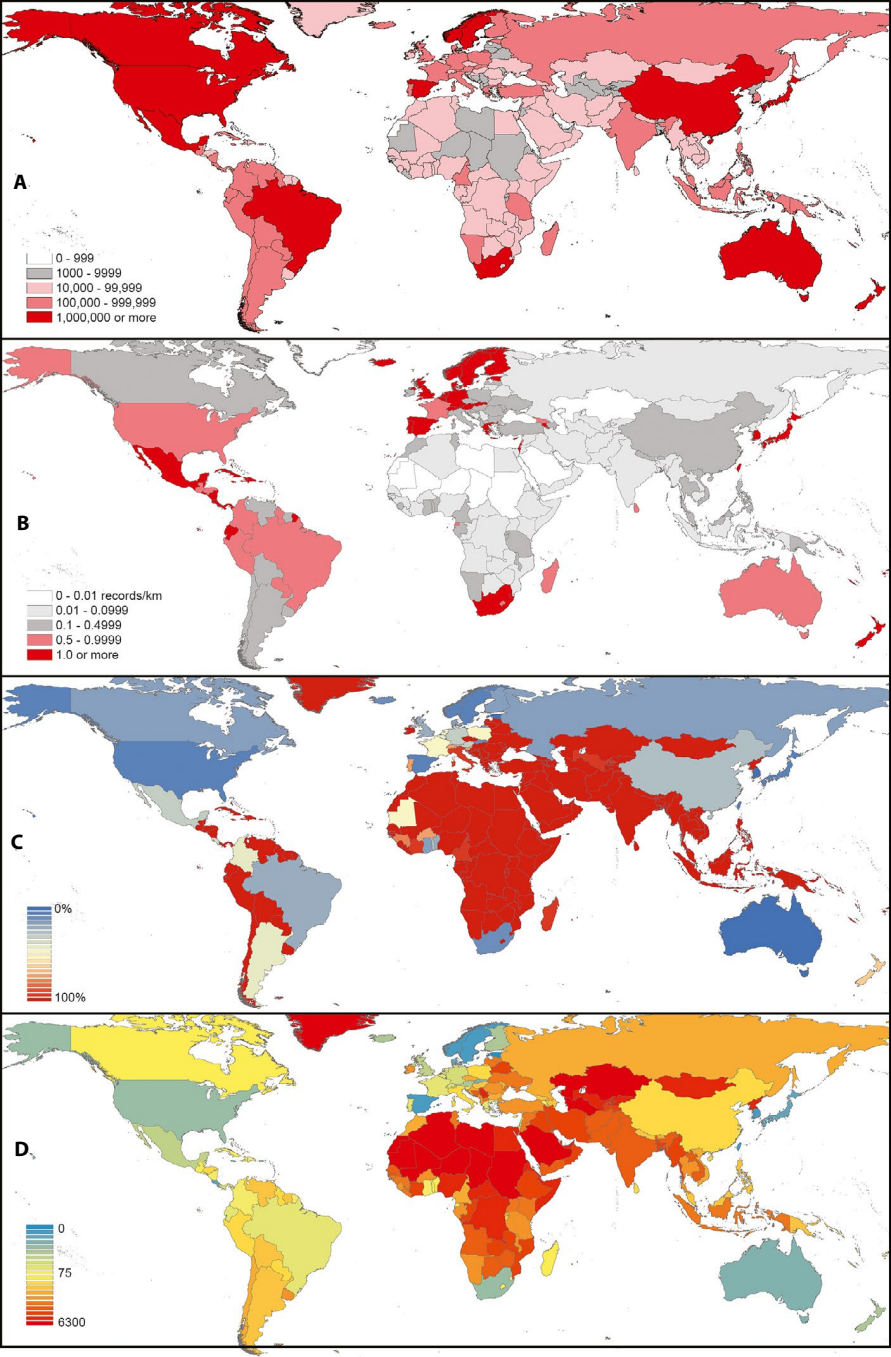
Four aspects of a global view of the density of digital accessible knowledge based on plant specimens. (A) Number of records in terms of raw numbers. (B) Record density on a per‐km^2^ basis. (C) 100× the proportion of a country's plant records that are made accessible by a different country. (D) Ratio between percent external and record density as an index to the need of a given country for mobilization of data records from outside countries.

In broad terms, then, the WAP Initiative generated >190,000 primary biodiversity occurrence data records in a span of two years. Presently, 351,700 such plant herbarium sheet data records are available from the region in the form of digital accessible knowledge records via GBIF (query available for replication at https://doi.org/10.15468/dl.yjr4tf [Global Biodiversity Information Facility, 2017b]); therefore, the WAP Initiative has increased available digital accessible knowledge records for West African plants by about 54%. Perhaps most significantly, the average cost of data capture in this project ranged from US$0.50–1.00/herbarium sheet for West African herbaria, but only US$0.50/herbarium sheet for images supplied to the project by European institutions. The average cost of data capture per specimen includes costs associated with tasks across the entire digitization workflow (e.g., preparation tasks, staff allowance, post‐digitization tasks). Finally, our efforts to identify regions with data to be captured, improved, and mobilized by those who wish to have access to plant biodiversity data emphasize much of Africa, the Middle East, and Central Asia, with South Asia and Southeast Asia not far behind (Fig. 5, bottom panel).

## DISCUSSION

### Antecedents

This project is a direct intellectual descendant of the monumental efforts by the Comision Nacional para el Conocimiento y Uso de la Biodiversidad (CONABIO) in Mexico. CONABIO, initiated in 1992, faced a difficult challenge: to be a reliable source of authoritative biodiversity data for the Mexican government (as well as for its scientific community) when the data were not organized or digitized at the time of CONABIO's founding. This was particularly challenging because collections digitization was still in its infancy and no open biodiversity information networks (e.g., VertNet, GBIF) yet existed (Canhos et al., 2004). CONABIO met this challenge by two main strategies: by funding data‐related biodiversity research and specimen curation and by sending out “data capture teams” to institutions around the world, particularly those that had no plans to initiate their own data capture. Data were captured by hand in most cases, sometimes with specimen‐by‐specimen capture, and in other cases based on images or photocopies of catalogs or specimen labels (Navarro‐Sigüenza et al., 2002, 2003). Although many of the current tools did not yet exist (e.g., DarwinCore [Wieczorek et al., 2012], capture interfaces involving images [Nelson et al., 2012], and automated georeferencing [Guralnick et al., 2006]), this work proceeded specimen by specimen and resulted in numerous comprehensive, large‐scale data resources for CONABIO and for the Mexican science and policy community (CONABIO, 2012).

Today, thanks to those multifaceted efforts by numerous taxon‐based teams, CONABIO has placed Mexico among the global leaders in biodiversity informatics and biodiversity information availability, such that the country's knowledge of its biodiversity‐related natural resources is equal to that of countries with greater financial resources. These information resources have permitted a meteoric increase in the quantity and quality of the biodiversity science developed in Mexico (Peterson et al., 2016a), as well as a key role for CONABIO in guiding and informing natural resource policy across Mexico and the Mexican government (CONABIO, 2012). To a more limited extent, other regions and taxonomic specialist communities have experimented with need‐based biodiversity information capture. For example, for Brazil's Reflora Project, the Paris and Kew herbaria provided large numbers of images of Brazilian herbarium specimens to the Brazilian team, which captured the associated data and provided high‐quality data in return, thereby creating exciting new, large‐scale information resources (Pignal et al., 2012; Sousa‐Baena et al., 2013). These antecedent efforts proved the concept to which we have adhered in the WAP Initiative: biodiversity data can be captured and improved efficiently and effectively when those who lead the initiative are precisely those who dream of having such data resources available.

### Was the West African Plants Initiative successful?

Biodiversity information for the West African region has heretofore been characterized by the existence of major gaps across geography (Koffi et al., 2015; Asase and Peterson, 2016; Ganglo and Kakpo, 2016) and, more broadly, across Africa (Dauby et al., 2016; Sosef et al., 2017). In this sense, the WAP Initiative has made major and significant progress, adding >190,000 new biodiversity records to the information available for the plants of the region. We note that capture of existing data, as prioritized in this project, makes considerable sense: (1) it is cost‐effective because it is much less expensive than de novo field collections, (2) it allows development of information resources even for regions in which political situations make contemporary field sampling impossible, and (3) it provides a historical baseline against which to compare newer data as the latter become available (Peterson et al., 2016b).

A particularly important dimension in which the WAP Initiative has been successful is that of being cost‐effective. A recent publication (Peterson and Soberón, 2017) presented data on costs of data resources developed by CONABIO over a large sample of projects and demonstrated a clear negative relationship between cost per record and overall magnitude of the project. It also showed that costs involved in creating CONABIO's data resources could be quite high, only rarely going below about US$5.00 per data record. In this initiative, we had per‐record costs of US$0.50–1.00.

Further measures of success will center on whether the institutions “owning” the specimens follow through and put the new data records online when the newly captured data are sent to the institutions where the specimens are located. Already, several project institutions have put initial project data online as part of their GBIF data contributions, but success should be defined as *all* project‐generated data being available online. Only when such data are fully online can they be applied in scientific analyses, in tandem with data provided by the same and other institutions under the aegis of other initiatives and/or institutional efforts.

Finally, we consider the West African Plants Initiative to have been a significant success because it has demonstrated the collective, emergent properties of broad, international collaborations. In this project, botanists from four West African countries cooperated and coordinated closely to generate a product that is more significant to science than any could have generated alone. The West African team has in turn coordinated efforts with institutions in Europe and North America, receiving significant and invaluable support from European herbaria in terms of large stores of images of herbarium sheets from across the West African region.

### Other venues and steps forward

Building and enhancing capacity in biodiversity informatics among West African students and scientists is critically important to effective and efficient data mobilization, data sharing, and data use. Abundant new biodiversity data may prove of little importance to the science infrastructure of the region if not accompanied by new scientific talent that allows the data to be put to good use. Several of our project participants have already initiated further studies to fill this personnel gap, but support for their studies and support for institutions to receive them upon completion of their studies are still uncertain.

Once captured, primary biodiversity data (as in this project) must be cleaned and georeferenced to yield high‐quality data resources. Such data will have many immediate applications such as documenting basic biodiversity patterns (Arita et al., 2008), identifying priority areas for conservation efforts (Loyola et al., 2007), detecting biotic change (Peterson et al., 2015), anticipating biotic responses to local and global change (Kearney et al., 2010), and guiding sustainable development and decision‐making about natural resources and the environment (Chapman, 2005; Sousa‐Baena et al., 2013). As such, skills development in data management and data improvement are key to improving captured data and developing high‐quality botanical information resources. A further need is skills in data analysis, such as in multivariate statistics, place‐prioritization efforts, and ecological niche modeling; such skills are necessary if mobilized data are to be used to inform national and regional decision‐making (see tools presented in Peterson and Ingenloff, 2016).

The scope of this particular project can and should be expanded, in terms of both West African and European/North American institutions, to improve geographic coverage and record density. Such an expansion in participation will also enhance the georeferencing process, because national scientists presumably have better knowledge of the geography of their own countries. West African countries such as Senegal, Guinea, and Burkina Faso have significant in‐country collections, and all West African countries have significant representation in collections in European and North American institutions for which data have yet to be captured. Significant holdings known to us include the herbaria of the Natural History Museum (London), the Smithsonian Institution, and Harvard University, and we hope that these institutions will participate in future chapters of this initiative.

### A new model for biodiversity informatics progress

This paper outlines, illustrates, and expands on what can be a new model for progress in biodiversity informatics, particularly for developing countries that are often rich in biological diversity but poor in biodiversity information. The quandary is that scientists and policy‐makers in those developing countries are often highly motivated and active in efforts to develop information resources, yet data are too frequently not available or not abundant; starting from zero with new sampling is not possible owing to cost, logistical considerations, land use change, and/or politics. However, institutions in Europe or North America, and sometimes elsewhere, often hold specimens with rich information coming from those same countries, but often have not captured those data completely, leaving information dormant and unavailable for applications in science, conservation, and natural resources policy.

In the solution that we explore herein, the scientists and policy‐makers in the countries and regions of origin of the biodiversity data are those who motivate the capture of the biodiversity data from their countries and regions. Institutions holding data and specimens continue to hold those materials and to curate and care for them, as they have in many cases for more than a century, assuring permanency of the resources. These specimen‐rich institutions provide access to persons and institutions from the regions of origin to promote their digitization. All involved—both the countries of origin and the institutions holding the specimens—cooperate in refinement and “publication” of the resulting data, and in the scientific interpretation and analysis of the new data resources.

Note that this model is the reverse of the traditional model, in which the institutions holding the specimens create the information resources that are used by the rest of the world. Here, instead, the motivation and implementation is carried out by scientists often based in the developing world, which heretofore have been rich in biodiversity but poor in biodiversity information. Given dramatic processes of globalization of scientific knowledge and ability (Dong et al., 2017), this new model has considerable promise to accelerate and improve the process of generating biodiversity information.

## Data Availability

The data resources generated by this project are available as online resources via the Global Biodiversity Information Facility (https://doi.org/10.15468/e8rhqm [Asase, 2018]; https://doi.org/10.15468/9czcig [Ugbogu et al., 2019]). Other data sets, yet to be published, are available from the authors upon reasonable request.
